# Superoxide dismutase 1 encoding mutations linked to ALS adopts a spectrum of misfolded states

**DOI:** 10.1186/1750-1326-6-77

**Published:** 2011-11-17

**Authors:** Mercedes Prudencio, David R Borchelt

**Affiliations:** 1 Department of Neuroscience, McKnight Brain Institute, University of Florida, Gainesville, FL 32610, USA; 2 Department of Neuroscience, Mayo Clinic College of Medicine, 4500 San Pablo Rd, Jacksonville, FL 32224, USA

## Abstract

**Background:**

Mutations in superoxide dismutase 1 (SOD1), which are one cause of familial amyotrophic lateral sclerosis (fALS), induce misfolding and aggregation of the protein. Misfolding can be detected by the binding of antibodies raised against peptide epitopes that are normally buried in the native conformation, shifts in solubility in non-ionic detergents, and the formation of macromolecular inclusions. In the present study, we investigate the relationship between detergent-insoluble and sedimentable forms of mutant SOD1, forms of mutant SOD1 with aberrantly accessible epitopes, and mutant protein in inclusions with the goal of defining the spectrum of misfolded states that mutant SOD1 can adopt.

**Results:**

Using combined approaches in cultured cell models, we demonstrate that a substantial fraction of mutant SOD1 adopts a non-native conformation that remains soluble and freely mobile. We also show that mutant SOD1 can produce multimeric assemblies of which some are insoluble in detergent and large enough to sediment by ultracentrifugation and some are large enough to detect visually. Three conformationally restricted antibodies were found to be useful in discriminating mal-folded forms of mutant SOD1. An antibody termed C4F6 displays properties consistent with recognition of soluble, freely mobile, mal-folded mutant SOD1. An antibody termed SEDI, which recognizes C-terminal residues, detects larger inclusion structures as well as soluble misfolded entities. An antibody termed hSOD1, which recognizes aa 24-36, detects an epitope shared by soluble non-natively folded WT and mutant SOD1. This epitope becomes inaccessible in aggregates of mutant SOD1.

**Conclusions:**

Our studies demonstrate how different methods of detecting misfolding and aggregation of mutant SOD1 reveal different forms of aberrantly folded protein. Immunological and biochemical methods can be used in combination to detect soluble and insoluble misfolded forms of mutant SOD1. Our findings support the view that mutant SOD1 can adopt multiple misfolded conformations with the potential that different structural variants mediate different aspects of fALS.

## Background

One consequence of fALS associated mutations in SOD1 [Swiss-Prot: P00441] that seems to be shared by all mutants is that the mutant SOD1 is far more prone to adopt aberrant conformations that result in its aggregation [1]. This common property is easily assessed in cultured cell models in which mutant SOD1 is overexpressed. However, pathologic evidence of mutant SOD1 aggregation in disease has also been consistently demonstrated in human fALS patients harboring mutations in SOD1 and transgenic mouse models of this disease. In both cases, there are multiple reports of the detection of SOD1 immunoreactive inclusions in spinal motor neurons [2-13]. For example, in transgenic mouse models of SOD1-associated ALS, SOD1 antibody reactive inclusions have been detected in spinal motor neurons of mice expressing the H46R [14], G85R [4,10], and G93A [7,8,10,13] mutants; and in spinal astrocytes of mice expressing the G85R [4] mutant. However, there have been reports of poor detection of SOD1-immunoreactive inclusions in spinal motor neurons of G37R, G85R, G93A, H46R/H48Q and Quad SOD1 transgenic mice [10,15,16]. Moreover, in mice expressing the G37R and G93A mutants at levels high enough to cause paralysis in 4 to 6 months, the more obvious pathology identified by SOD1 antibodies is a vacuolar pathology [15,17]. In general, in the aforementioned studies in which SOD1-immunoreactive inclusions have been detected, they have generally been found to be most abundant in end-stage mice.

Misfolded mutant SOD1 can also be detected biochemically, using an assay that involves detergent extraction and centrifugation followed by western blotting [1,18-21]. In transgenic mouse models of SOD1-fALS, detergent insoluble forms of mutant SOD1 accumulate to high levels late in the course of disease, becoming readily detectable at the onset of visible symptoms [22]. In the G93A-Gur1 model, which reaches end-stage paralysis at about 120 days, aggregates begin to accumulate between 90 and 105 days and then rise dramatically as the animals approach endstage [22]. During this same interval the levels of neurofilament H in serum, which serves as a biomarker of axonal degeneration [23], also rise dramatically. Thus we can correlate aggregation of mutant SOD1 and axonal degeneration in the animal models. However, it remains difficult to assign cause and effect as it is possible that the accumulation of aggregates serves as a biomarker of cellular degeneration in which the cells simply lose the ability to prevent the misfolded mutant protein from aggregating because of some combination of declining chaperone activities, declining proteasome activity to degrade the misfolded protein, or declining energy production to support these activities. Moreover, in the best studied G93A model, it is very clear that numerous pathologic abnormalities can be detected prior to the accumulation of aggregated mutant SOD1, indicating that other misfolded forms of mutant SOD1 must mediate early events in the evolution of disease [22].

One method that has emerged as a means to specifically detect misfolded forms of mutant SOD1 involves SOD1 antibodies raised against specific peptide sequences that are normally buried in the tertiary structure of native WT protein or against preparations of mutant SOD1 [17,24,25]. Some of these antibodies have the potential to detect a variety of structural variants of SOD1, including immature unfolded protein, misfolded soluble protein, and inclusion structures. In a study of the G93A mouse model, Rakhit et al [17] described an antibody to sequences in the C-terminus of SOD1, called the SEDI antibody, which showed minimal reactivity with natively folded WT SOD1. As compared to tissues from mice expressing WT SOD1, tissues from symptomatic mice expressing mutant SOD1 (G37R, G85R, and G93A) contained higher levels of SOD1 proteins that reacted with the SEDI antibody. In tissue sections from these animals, the SEDI antibody stained SOD1, accumulating at the rims of vacuolar pathology in the G37R and G93A mice (more rarely inclusion like structures were stained) and structures resembling inclusions in the G85R mice (vacuolar pathology limited in this model). In presymptomatic G37R and G93A models, SEDI immunoreactivity was abundant at the rims of vacuolar pathology that is among the first identifiable pathologic abnormality, occurring well before the onset of symptoms [17]. Collectively, these studies with conformationally-restricted antibodies indicate that misfolded SOD1 proteins are present early in the evolution of disease.

In the present study, we have sought to use cell culture models to investigate the characteristics of the various forms of misfolded mutant SOD1 proteins and to investigate the relationships between misfolded SOD1 proteins, detergent insolubility, and inclusions. To accomplish this goal, we combine the visual capability of the YFP fusion proteins with biochemical assays of insolubility and with immunohistochemical approaches using conformationally restricted antibodies. Our findings provide new insight into the spectrum consequences that fALS mutations have on the folding of SOD1 and how different approaches to detection can be used to reveal aspects of mutant SOD1 misfolding.

## Results

### Visualization of misfolded and aggregated mutant SOD1 in cultured cell models

**Figure 1:**
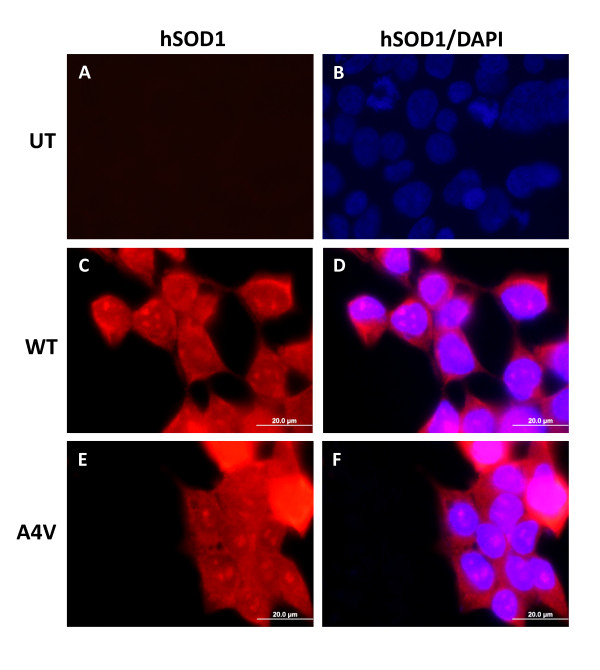
**HEK293FT cells expressing mutant SOD1 proteins do not form visible cellular inclusions**. A-I) Cells were cultured on glass coverslips previously coated with 0.5 mg/ml poly-L-lysine untransfected (A, B), or transfected with expression plasmids for WT (C, D) or A4V (E, F) SOD1 for 24 hours. Fixed cells were stained with hSOD1 antibody overnight. A secondary fluorescent (594 nm) antibody was used to visualize SOD1 antibody binding. Co-staining with 4',6-diamidino-2-phenylindole (DAPI) was performed together with secondary antibody incubations. Pictures were taken with a 100× immersion oil objective, scale bar = 20 μm. Note that at the same exposure, the staining of overexpressed hSOD1 protein is much brighter than that of untransfected cells (compare figure A to C and E). The images shown are representative of at least 3 independent transfection experiments.

The A4V mutation in SOD1 is associated with rapidly progressing disease and when expressed in HEK293FT cells is among the most prone to produce detergent-insoluble aggregates of mutant protein [1]. To date, however, we have not sought to visualize whether the accumulation of detergent-insoluble aggregates is accompanied by inclusion formation in these cell models. Following previously established protocols [1,20,21], HEK293FT cells were transiently transfected with expression vectors for WT and A4V mutant SOD1, for 24 hours, then fixed and analyzed by immunofluorescence staining with the hSOD1 antibody, which was raised against a synthetic peptide corresponding to residues 24-36 and which specifically recognizes human SOD1 protein (hSOD1 antibody) [4]. This antibody cannot immunoprecipitate natively folded, fully metallated, WT SOD1 protein and is thus conformationally restricted (see Supplemental Material in [26]). Compared to untransfected cells (Figures 1A and 1B), cells expressing either WT (Figures 1C and 1D) or A4V SOD1 (Figures 1E and 1F) showed diffuse immunofluorescence without obvious cytoplasmic inclusions. Within nuclei of cells transfected with either WT or mutant SOD1, there were punctate structures that appeared to be some concentration of SOD1 in or around nuclear structures. The significance of these structures is uncertain at present.

In these experiments, the absence of immunoreactivity by the hSOD1 antibody in the untransfected human HEK293FT cells may be due partially to relative levels of protein; in the transfected cells the expressed SOD1 protein is much more abundant and thus at exposures optimal for visualization of the expressed protein, untransfected cells were virtually invisible. Additionally, we expect the endogenous protein to be more fully metallated and thus less reactive to the hSOD1 antibody [26]. Therefore, we primarily visualized the over-expressed SOD1 proteins in these experiments.

In detergent extraction of cells transfected in parallel, we observed, as previously reported [1,16], an accumulation of detergent-insoluble mutant protein (Additional File 1, Figure S1). However, only a minority of the total A4V SOD1 protein that was expressed in these cells was insoluble in detergent (Additional File 1, Figure S1). Thus, we expected that either most cells would show diffuse staining with only a minority having enough expression to induce inclusion formation, or that there might be small visible inclusions within a general background of diffusely distributed mutant protein. The absence of inclusion detection could also be due to inaccessibility of the antibody to its target epitope. As noted above, the hSOD1 antibody cannot immunoprecipitate natively folded WT SOD1, but shows strong reactivity to denatured protein (see Supplemental Material in [26]). Therefore, we asked whether treating the cells with 70% formic acid to denature proteins would reveal hSOD1 immunoreactive inclusions (Additional File 1, Figure S2). However, we still failed to detect inclusions.

To determine whether we might have better detection of inclusions in mouse cells, using the human SOD1 specific hSOD1 antibody, we transfected mouse fibroblast cultures with the same constructs and immunostained with the same antibody (Additional File 1, Figure S3). However, we observed the same diffuse immunostaining pattern that was observed in the HEK293FT cells.

**Figure 2:**
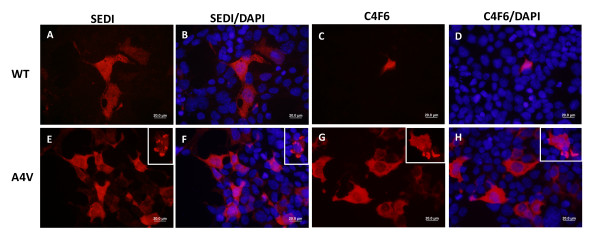
**SEDI and C4F6 antibodies recognize diffusely distributed cytosolic mutant SOD1 in transiently transfected HEK293FT cells**. Cells were transiently transfected with expression plasmids for WT (A-D) or A4V (E-H) SOD1 for 24 hours and stained with SEDI or C4F6 antibodies as explained in Methods. Pictures were taken with a 40× objective. Scale bars = 20 μm. Inset, image of rare cell with inclusion like structures. The images shown are representative of at least 3 independent transfection experiments.

We next examined HEK293FT cells expressing WT and mutant A4V SOD1 proteins by immunostaining cells with additional SOD1 antibodies that have been described as conformationally-restricted in binding SOD1 protein. One antibody used was the SEDI SOD1 antibody, which recognizes amino acids 143 to 151 in the dimer interface of SOD1; these residues are normally buried within the native protein [17]. Another antibody used was the C4F6 antibody, which was raised against the human G93A variant of SOD1 and which has been described to have very low immunoreactivity to native WT SOD1 in favor of mutant SOD1 [27]. For WT SOD1 expressing cells, SEDI immunoreactivity was generally much lower, although a few cells were found to be highly reactive (Figures 2A and 2B). Similarly, cells expressing WT SOD1 showed limited reactivity with the C4F6 antibody (Figures 2C and 2D). In cells expressing A4V hSOD1, immunoreactivity to SEDI largely appeared as uniform fluorescence; similar to the few immunoreactive WT expressing cells (Figures 2E and 2F). Rarely, we were able to find what appeared to be inclusion structures (Figure 2E and 2F, inset). Immunostaining of cells expressing A4V hSOD1 with the C4F6 antibody gave images similar to those observed with SEDI antibody (Figures 2G and 2H), infrequently finding cells that contained intracellular inclusion-like structures (Figures 2G and 2H, inset). Overall, regardless of which antibody was used, immunoreactive inclusion structures were rare in cells expressing untagged A4V hSOD1. As noted above, the majority of SOD1 in cultures expressing A4V SOD1 was soluble in detergent (see Additional File 1, Figure S1). Thus, these cells accumulate a form of mutant SOD1 that is soluble in non-ionic detergents and yet is reactive to antibodies that bind epitopes normally buried in the native conformation of WT SOD1.

### Relationship between inclusions and detergent-insoluble SOD1

**Figure 3:**
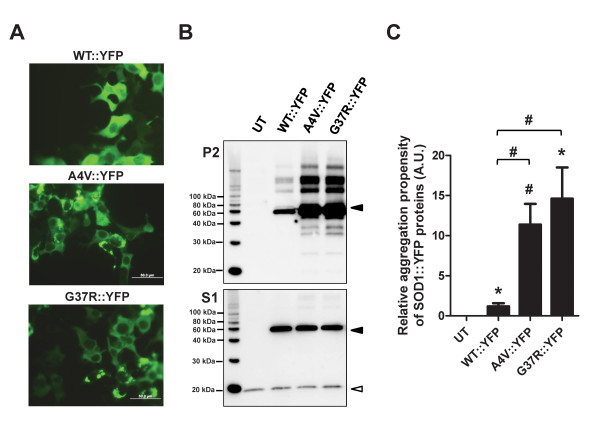
**Mutant SOD::YFP proteins form visible inclusions in cell culture**. A) HEK293FT cells, on poly-L-lysine coated glass coverslips, were transfected for 24 hours as described in Methods. Cells were fixed and observed under a fluorescence microscope. All pictures were taken using a 40x objective, scale bars = 50 μm. The images shown are representative of at least 3 independent transfections for each construct. B) Immunoblot of P2 and S1 protein fractions of cells expressing SOD1::YFP proteins for 24 hours. The SOD1::YFP protein migrates at a size of approximately 50 kDa (filled arrowhead), while endogenous WT SOD1 monomer runs at 16 kDa (open arrowhead). C) Quantification of the aggregation propensity (P2/S1) of cells expressing SOD1::YFP proteins (see Methods). As expected, cells transfected with WT::YFP or mutant SOD1::YFP accumulated much more insoluble protein than untransfected cells: *p ≤ 0.05; #p ≤ 0.005. The aggregation propensity of A4V::YFP and G37R::YFP were both statistically greater than that of WT::YFP (#p ≤ 0.005). The data were averaged from at least 3 independent repetitions of each transfection and immunoblot.

To more easily visualize which cells express mutant SOD1 and which cells develop inclusions, we generated variants of SOD1 tagged with yellow fluorescent protein (YFP). Multiple laboratories have used similar approaches to visualize inclusions formed by mutant SOD1 in living cells using fluorescent proteins such as green fluorescent protein (GFP), YFP, red fluorescent protein (RFP), or DsRed2 in various cultured cell models [28-35]. Thus we created several SOD1::YFP constructs to express in cultured cells as a means to analyze the relationships between detergent-insoluble aggregates that can be detected biochemically and visible cellular inclusions. We used two cell models for these studies; the previously described HEK293FT cell model [1,20,21] and Chinese Hamster Ovary cells (CHO cells). Transient transfections of WT SOD1 tagged with YFP (WT::YFP) in HEK293FT for 24 hours showed uniform distributions of fluorescence throughout the cytosol (Figure 3A). SOD1::YFP fusion proteins generated from the A4V and G37R variants of SOD1 (A4V::YFP and G37R::YFP) frequently produced cytosolic inclusion-like structures (Figure 3A). Notably, not all cells produced inclusions and there were many examples of cells with diffusely distributed YFP fluorescence. This outcome may be explained by the non-homogenous expression of transiently transfected cells; we presume, but cannot definitively prove, that only the cells with very high levels of expression develop inclusions. The appearance of inclusions was accompanied by the accumulation of significant amounts of detergent-insoluble mutant fusion proteins (Figure 3B). Notably, cells expressing WT::YFP SOD1 also accumulated significant amounts of detergent-insoluble fusion protein. However, the relative aggregation propensity of WT::YFP SOD1 protein was much lower than that of the mutants (Figure 3C). We observed a similarly high frequency of inclusions in cells expressing other fALS mutants of SOD1 and YFP (G85R::YFP, D101N::YFP, S134N::YFP), with similar accumulations of detergent insoluble fusion protein (Additional File 1, Figure S4).

**Figure 4:**
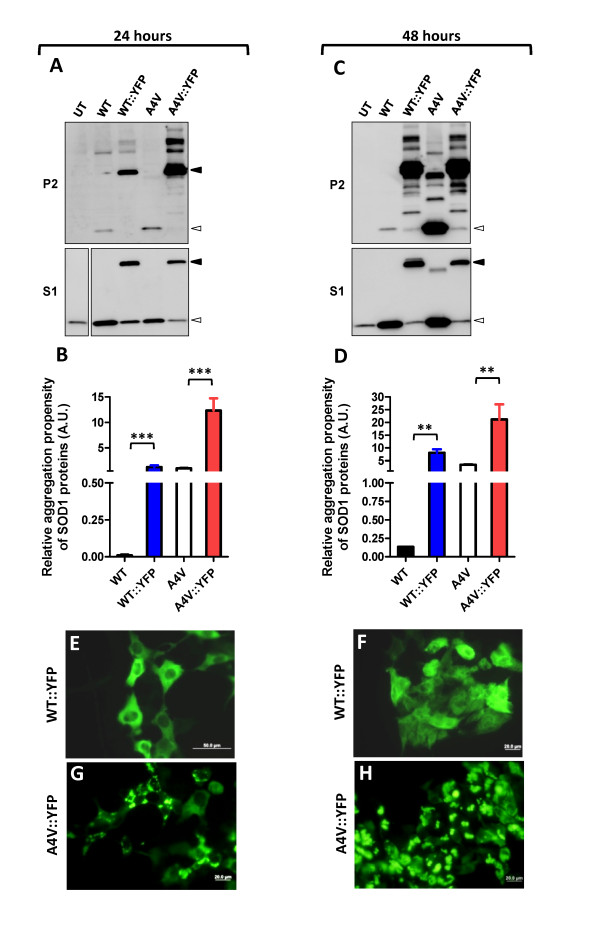
**Analysis of detergent solubility and inclusion formation in cells co-expressing WT and mutant SOD1**. HEK293FT were transfected with the indicated SOD1 constructs for 24 (A, B, E, G) or 48 (C, D, F, H) hours as noted above the figure. A, C) Immunoblots of P2 and S1 fractions of HEK293FT cells co-transfected with the indicated SOD1 constructs for 24 (A) or 48 (C) hours and immunoblotted with a SOD1 antibody. Tagged SOD1::YFP proteins migrate on SDS-PAGE at relative molecular mass of 50 kDa, while untagged SOD1 variants migrate at about 16 kDa. B, D) Quantification of the aggregation propensities (P2/S1) of tagged and untagged proteins from singly or doubly transfected cells at 24 (B) and 48 (D) hours. Statistical comparisons were calculated through unpaired student t-tests, *p ≤ 0.05, #p ≤ 0.005. The data were averaged from at least 3 independent transfection and immunoblotting experiments. E-H) Cells transfected for 24 (E, G) or 48 hours (F, H) were fixed and visualized under a confocal microscope. Scale bars = 20 μm. The images shown are representative of at least 3 independent transfection experiments.

To examine the relationship between inclusion formation and the formation of detergent-insoluble structures, we compared the levels of insoluble untagged SOD1 and SOD1::YFP fusion proteins that accumulate in our transfected cells (Figure 4). As compared to cells expressing WT SOD1, cells expressing WT::YFP SOD1 fusions accumulated significantly higher levels of insoluble SOD1 at 24 (Figures 4A and 4B) and 48 (Figures 4C and 4D) hours after transfection. However, despite the accumulation of such large amounts of detergent-insoluble SOD1 we did not observe inclusion structures in cells expressing WT::YFP fusions (Figures 4E and 4F). By contrast, in cells expressing A4V::YFP fusions, inclusion structures were visible in 24 hours, becoming more abundant at 48 hours (Figures 4G and 4H). Relative to WT::YFP fusions, the level of insoluble protein (ratio of insoluble to soluble) in cells expressing A4V::YFP fusions was substantially higher at both 24 and 48 hours. Still, the amount of detergent-insoluble WT::YFP fusion protein that accumulated at 48 hours was similar to cells expressing A4V::YFP fusion for 24 hours (Figures 4A-D) but only the latter was able to form numerous visible inclusions (Figures 4G and 4H). We interpret this outcome as evidence that the WT::YFP SOD1 fusion proteins are capable of forming detergent-insoluble structures that are distinct from insoluble inclusions.

### Conformationally restricted antibodies to SOD1

**Figure 5:**
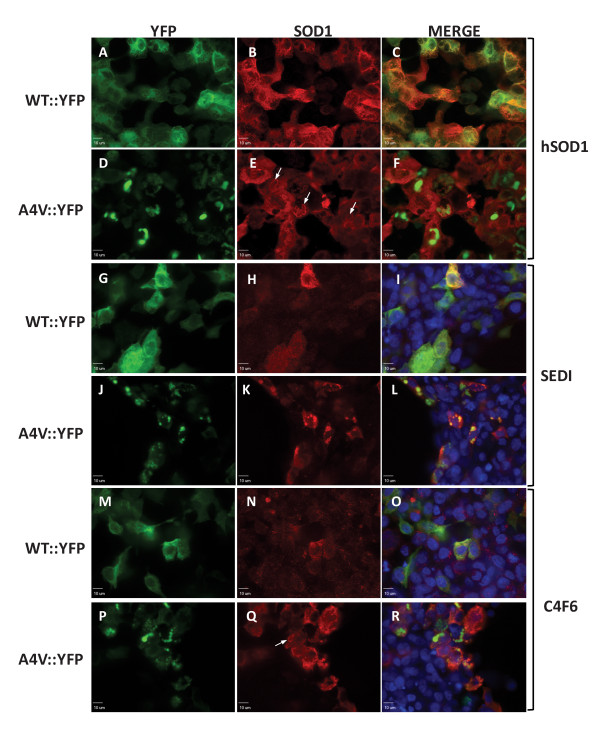
**Immunostaining of cells expressing SOD1::YFP fusion proteins with conformationally restricted SOD1 antibodies**. HEK293FT were transfected with WT::YFP or A4V::YFP constructs, as noted on the Figure, and stained with hSOD1 (A-F), SEDI (G-L) or C4F6 (M-R) antibodies as explained in Methods. Pictures were taken using a 60× water immersion objective of a spinning disc confocal microscope, scale bars = 10 μm. Arrows indicate the location of SOD1::YFP inclusions and the absence of antibody staining. The images shown are representative of at least 3 independent transfection experiments.

With the ability to easily visualize which cells express mutant SOD::YFP fusion proteins and which cells produce inclusions, we sought to determine the efficacy with which different conformationally restricted SOD1 antibodies detect inclusions. For this study, we used the same three antibodies described above (hSOD1, SEDI and C4F6). WT::YFP SOD1 expressing cells immunostained with hSOD1 antibody showed uniform cytosolic distribution (Figures 5A-C), indicating a portion of the expressed protein did not achieve a native conformation. In cells expressing A4V::YFP proteins, we observed the formation of inclusions containing A4V::YFP SOD1, however the hSOD1 antibody appeared to recognize only diffusely dispersed A4V::YFP protein (Figures 5D-F). Although hSOD1 did not appear to directly detect inclusion structures, we could identify several features in which it appeared that hSOD1 antibody recognized protein that appeared to be concentrated around the surface of the inclusion (Figure 5E, arrows). Thus, we conclude that the sequence recognized by the hSOD1 antibody (residues 24-26) is inaccessible in mutant SOD1 that is compartmentalized in inclusion structures.

The SEDI and C4F6 antibodies also recognized diffusely distributed SOD::YFP proteins in the cytosol, including, more infrequently, WT::YFP fusion proteins (Figures 5G-I, and 5M-O). In cells expressing A4V::YFP, the SEDI antibody was highly reactive with inclusion-like structures (Figures 5J-L). The C4F6 antibody showed moderate reactivity with diffusely distributed WT::YFP fusion protein in a small subset of cells (Figures 5M-O), but showed strong reactivity to diffusely distributed A4V::YFP fusion protein (Figures 5P-R). We did not observe immunostaining of inclusions with the C4F6 antibody, thus the epitope recognized by C4F6 antibody is normally buried when the mutant SOD1::YFP proteins form inclusion structures whereas the epitope of the SEDI antibody is exposed.

### Mutant SOD1 inclusions and detergent-insoluble SOD1 are not freely mobile in cytosol

**Figure 6:**
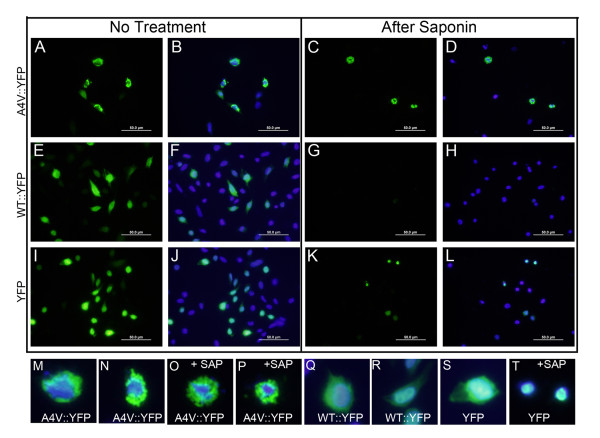
**Inclusions formed by mutant SOD1::YFP proteins are not released by saponin treatment**. CHO cells were transiently transfected with expression plasmids for YFP, WT::YFP, and A4V::YFP as noted in the figure and then, after 24 hours, treated with saponin, fixed in 4% paraformaldehyde, stained with DAPI, and imaged. A, B, E, F, I, J, M, N, Q, R, and S were not treated with saponin. C, D, G, H, K, L, O, P, and T were treated with saponin. Scale bars in A-L are 50 μm. The images in M-T are digitally magnified cells from A-L. Because saponin completely extracted all cyotosolic fluorescence from cell expressing WT::YFP, no digital magnifications of these cells are shown. The images shown are representative of at least 3 independent transfection experiments. Scale bar = 50 μm. Images were capture on a standard fluorescence microscope.

A previous study by Urushitani et al examined mouse N2a cells expressing mutant SOD1-EGFP fusion proteins, finding that digitonin released most of the expressed protein but in cells expressing mutant SOD1-EGFP, punctate inclusion-like structures remained cell associated [34]. We applied the same technique to our cell models. Similar to digitonin, saponin is a mild detergent that open pores on the cellular membranes without completely lysing the cells, allowing soluble proteins to diffuse out of the cell [36]. In this experiment, CHO cells were used because these cells were less prone to dislodge after saponin treatment. Cells transfected with a vector for A4V::YFP fusion proteins produced characteristic punctate inclusions that were not released into culture medium by saponin (Figures 6A-D, and 6M-P). By contrast, WT::YFP fusion proteins showed a more diffuse localization (Figures 6E-H, and 6Q, R) and this fluorescence was completely released by saponin treatment (Figures 6G and 6H). Note that a significant fraction of WT::YFP SOD1, shown above in Figure 3, is insoluble in non-ionic detergent and yet virtually all of the fluorescence is released from these cells by saponin. In cells expressing YFP alone, fluorescence was similarly diffusely distributed with saponin releasing most of the fluorescence into the medium (Figures 6I-L, and 6S, T). However, some localization of the YFP to nuclei was observed and this nuclear protein was not released by saponin (Figures 6K, L, T). These findings indicate that the general distribution of inclusion structures does not obviously change with saponin treatment, suggesting the structures are bound to immobile elements within the cytosol [36].

**Figure 7:**
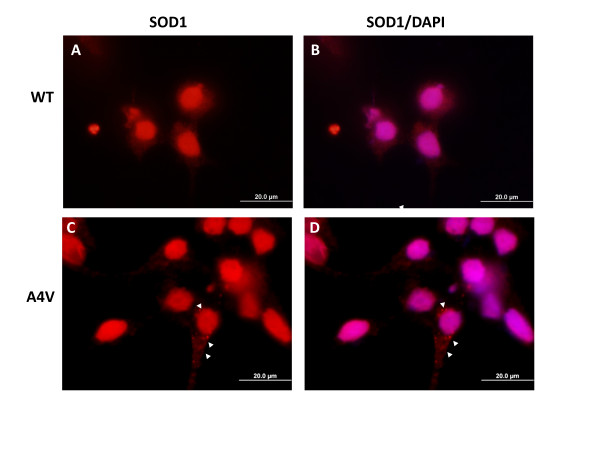
**Saponin treatment extracts cytosolic SOD1 immunostaining**. HEK293FT cells were transfected with vectors for WT (A, B) or A4V SOD1 (C, D) and after 24 hours treated with saponin for 30 minutes at a concentration of 0.01% in 1× PBS. The cells were then fixed and immunostained as explained in Methods. Images were captured on a standard fluorescence microscope with an immersion oil 100× objective. All bars represent 20 μm. The images shown are representative of at least 3 independent transfection experiments.

Based on the immobility of mutant SOD1::YFP inclusions, we once again revisited the detection of mutant SOD1 aggregates generated by untagged mutant protein in transfected HEK293FT cells by asking whether treatment of cells with saponin will unmask inclusions in cells expressing untagged A4V SOD1. Saponin treatment efficiently extracted nearly all of the cytosolic SOD1 immunoreactivity in cells expressing WT SOD1 (Figures 7A and 7B). The only remaining hSOD1 immunoreactive protein was concentrated in, or possibly around, the nucleus (Figures 7A and 7B). This latter finding is indicative of the small size of SOD1, which would facilitate passive diffusion into the nucleus [37], and is consistent with prior observations that SOD1 is found in nuclear compartments [38]. Despite removing most of the cytoplasmic immunoreactivity, it was still difficult to identify cells containing obvious inclusions in cells expressing A4V hSOD1 (Figures 7C and 7D). Very rarely we observed small punctate structures in a perinuclear location (Figures 7C and 7D, arrow heads).

We also investigated the expression of mutant human SOD1 in mouse TK-L cells, in which we could use antibodies specific for human SOD1 to potentially more specifically visualize inclusions formed by mutant human SOD1. However, after treatment with digitonin, we observed immunoreactive structures in cells transfected mutant hSOD1 that were similar to what we observed in cells expressing WT SOD1 (Additional File 1, Figure S5).

**Figure 8:**
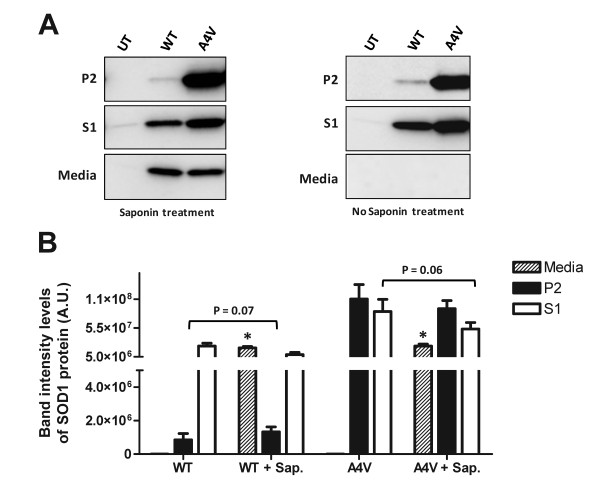
**Detergent-insoluble forms of mutant SOD1 remain cell associated after saponin treatment**. A) Immunoblots of HEK293FT cells that were transfected with the indicated SOD1 constructs for 48 hours. Prior to harvest, cells were incubated 30 minutes in PBS with 0.01% saponin. The harvested cell pellets were analyzed by detergent extraction and centrifugation assays (S1 fractions, each lane contains 5 μg of total protein; P2 fractions, each lane contains 20 μg of total protein). The amount of SOD1 protein released into the cell media was also evaluated (each lane loaded with 5 μg of total protein). B) Quantification of SOD1 protein found in the Media, P2 and S1 fractions of cells untreated or treated with saponin (Sap.), and expressing WT or A4V. Symbols over bars indicate differences from corresponding non-treated control. Unpaired *t*-tests: *p ≤ 0.05. The data were averaged from at least 3 independent transfection and immunoblotting experiments.

To verify that aggregated forms of untagged SOD1 were not released by saponin treatment, cells treated with saponin were subjected to detergent extraction and centrifugation assay before immunoblotting soluble and insoluble fractions (Figure 8), following previously described procedures [1,20,21]. As expected, saponin treatment induced the release of a significant amount of both WT and mutant SOD1 protein into the cell culture media (Figures 8A and 8B). In the case of cells transfected with WT SOD1, a substantial portion of SOD1 protein was released into the media with the fraction remaining with the cell exhibiting near complete solubility in detergent (Figures 8A and 8B). A minor fraction of the WT SOD1 was insoluble in detergent and there was no significant change in the solubility of WT SOD1 after saponin treatment (Figure 8B). In cells expressing A4V SOD1, the levels of SOD1 released by saponin were very similar to that of cells expressing WT SOD1 (Figures 8A and 8B). As was observed in cells expressing WT SOD1, there were still relatively high levels of detergent-soluble, cell-associated, A4V SOD1 after saponin treatment (Figure 8B). This detergent-soluble mutant protein may be protein that diffused into the nucleus. Importantly, in cells expressing the A4V mutant, saponin treatment did not significantly reduce the amount of detergent-insoluble mutant protein associated with the cells (Figure 8B). Similar outcomes were observed in cells treated with digitonin (Additional File 1, Figure S6), which similar to saponin, permeabilizes cell plasma membranes without completely lysing cells. These findings indicate that the majority of the insoluble mutant SOD1 in cells is immobilized by association with some cytosolic structure.

**Figure 9:**
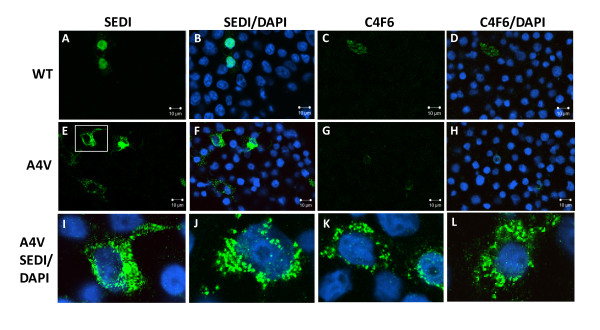
**The SEDI antibody recognizes immobile punctate structures in cells expressing A4V hSOD1**. HEK293FT cells were transiently transfected with expression plasmids for either WT (A-D) or A4V (E-L) hSOD1 for 24 hours and stained with the SEDI or C4F6 antibody as explained in Methods. Pictures were taken with a 40x objective. Scale bars = 10 μm. The images in I-L were digitally magnified from A4V SOD1 cells stained with SEDI/DAPI. The images shown are representative of at least 3 independent transfection experiments.

Based on the findings above, we were confident that a substantial portion of mutant SOD1 aggregates are not extracted by saponin treatment. Therefore, we applied these techniques to HEK293FT cells transfected with expression vectors for untagged SOD1. Twenty-four hours post-transfection, saponin-treated cells were fixed and immunostained with the SEDI and C4F6 antibodies (Figure 9). Neither antibody detected cytosolic reactivity in cells expressing WT hSOD1 (Figures 9A-D), indicating that the cytosolic immunoreactivity detected with this antibody (see Figures 1 and 2) is released into the medium by saponin. In cells expressing A4V hSOD1, the diffuse staining seen in untreated cells with both antibodies (see Figure 2) was also lost. Instead we were now able to clearly identify cells with punctate cytosolic structures that were reactive to SEDI antibody (Figures 9E-F and 9I-L). By contrast, the C4F6 antibody did not detect these punctate structures (Figures 9G and 9H). These findings were consistent with what was observed in cells expressing SOD1::YFP fusion proteins and indicate that the SEDI antibody is useful in detecting mutant SOD1 in inclusion structures.

### Mutant SOD1 aggregation and toxicity

**Figure 10:**
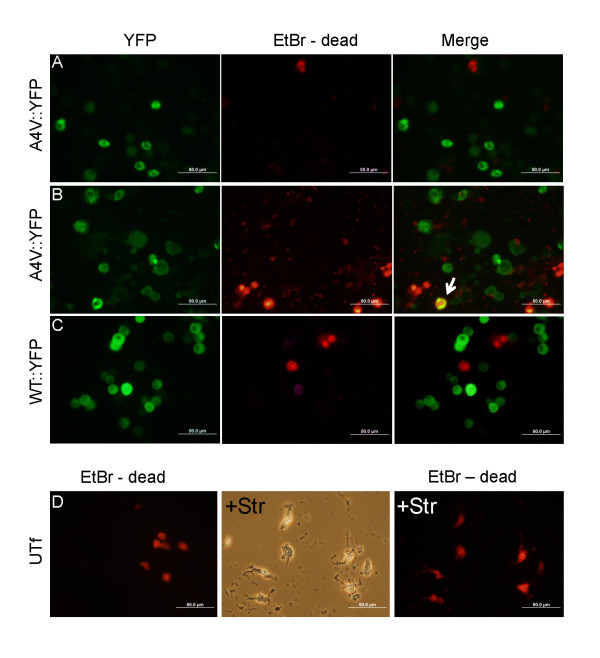
**CHO cells expressing A4V::YFP SOD1 fusion protein do not show evidence of increased membrane permeability**. To assess whether aggregates of mutant SOD::YFP fusion proteins induce cell death, CHO cells were transiently transfected with expression vectors for SOD1 A4V::YFP (A and B) and WT::YFP (C). After 48 hours, the cells were incubated with dimeric ethidium bromide (EtBr) as described in Methods. Images were captured at 40× magnification (scale bar = 50 μm). Overall, there were few cells that showed evidence of increase membrane permeability with no obvious difference between cultures transfected with the 2 constructs or untransfected cells (D). As a positive control, untransfected cells were treated with staurosporine (+Str) to induce death and stained with dimeric ethidium bromide (D).

Previous studies by Matsumoto et al [31], in a PC12 cell model of transient transfection, have demonstrated that cells harboring inclusions formed by YFP fusions of G85R and G93A SOD1 become permeable to propidium iodide (a reporter of cell death). We sought to determine whether our cell models of aggregation show evidence of toxicity, using two reporters systems of cell death. We initially used the CHO cells because we found these cells to be more suited to viewing individual cells; they were less prone to clumping, exhibited a flatter morphology, and showed better adherence (meaning fewer were lost during various steps in preparation). The YFP tagged variants of mutant SOD1 facilitate direct visualization of whether cells that harbor aggregates are undergoing degeneration. To assess whether cells expressing mutant or WT::YFP SOD1 fusion proteins might be dying, we used a cell death assay based on uptake of a homodimer of ethidium bromide. Cells that have lost membrane integrity take up the ethidium homodimer and fluoresce red upon binding of the ethidium to nucleic acids. We observed only low levels of cell death in cells expressing A4V::YFP SOD1 fusion proteins with similar levels occurring in cells expressing WT::YFP SOD1 fusion proteins and untransfected cells (Figure 10). Only rarely did we observe cells harboring A4V::YFP SOD1 inclusions showing uptake of ethidium homodimer. Importantly, in these studies, cells showing diffuse distributions of mutant or WT::YFP SOD1 fusion proteins also lacked evidence of obvious increases in uptake of the cell death marker. A similar outcome was obtained in the HEK293FT cell model (Additional File 1, Figure S7).

We also assessed whether cells with inclusion showed markers of apoptosis, using a TUNEL assay. For this experiment we used only the CHO cell model because the HEK293FT cells were so poorly adherent through processing steps that we could not make unbiased observations. At 48 hours post-transfection, we found that relatively few CHO cells were positive and only rarely did we observe TUNEL labeling of cells that expressed A4V::YFP SOD1 fusion proteins (Additional File 1, Figure S8). A similar low frequency of TUNEL positive cells was seen in cells expressing WT::YFP SOD1 fusion proteins and untransfected cells (Additional File 1, Figure S8). Overall, we observed no obvious overlap between cells expressing WT or A4V SOD1::YFP fusion proteins (whether inclusions were present or absent) and either marker of apoptosis or death. We conclude that these cells show limited toxicity in response to the expression of the fusion proteins.

## Discussion

In the present study, we use a combination of natural SOD1 mutants and SOD1::YFP fusion proteins with conformationally-restricted antibodies to examine the relationships between mutant SOD1 misfolding, aggregation, and inclusion body formation. Our findings are consistent with a scenario in which mutant SOD1 is capable of adopting a continuum of structures that include soluble non-natively folded proteins, detergent-insoluble multimeric complexes, and larger inclusion bodies. We observe that mutant SOD1 can adopt conformations that remain soluble in aqueous buffers and are freely mobile and yet are recognized by conformationally-restricted antibodies such as the SEDI and C4F6 antibodies. As previously described, we observe that mutant SOD1 adopts a conformation that promotes its assembly into larger structures that are not soluble in non-ionic detergents and sediment by centrifugation. In cells expressing untagged versions of mutant SOD1, using conformationally restricted antibodies, we found that the accumulation of detergent-insoluble protein correlated with the appearance of small punctuate structures that could be viewed as small micro-aggregates. These structures were recognizable by the SEDI antibody, but not by the C4F6 antibody. Moreover, although the accumulation of detergent-insoluble mutant protein correlated with the appearance of inclusion like structures for mutant SOD1, we found an example in which detergent-insolubility and inclusion formation was dissociated. Large amounts of detergent insoluble WT::YFP we detected in cells expressing this SOD fusion protein but inclusion structures were rarely visualized. Notably, this detergent-insoluble protein was, however, freely mobile and could be released by saponin. This finding indicates that SOD1 in detergent-insoluble conformations may not necessarily be assembled into large inclusion structures and can remain freely mobile. Thus, we demonstrate that mutant SOD1 has the capacity to produce a continuum of structures with each exhibiting features that are selectively revealed depending upon the detection methods used.

### SOD1 conformation and antibody epitope accessibility

In this report, we used three previously described antibodies that each show restricted reactivity to native conformations of SOD1. For the hSOD1 and SEDI antibodies, previous studies have demonstrated that these antibodies are not capable of immunoprecipitating natively folded WT SOD1 [17,26]. The C4F6 antibody has been shown to react poorly with natively folded WT SOD1 in immunoblots of non-denaturing SDS-PAGE [27]. The hSOD1 antibody, which was raised against specific peptide sequences of human SOD1 (residues 24-36), recognized soluble, freely mobile, forms of both WT and mutant SOD1. In work that will be published elsewhere, we have found that the vast majority of WT or mutant SOD1 that is over-expressed in these cells does not contain Cu (H. Lelie and D Borchelt, unpublished observation). We thus suggest that the lack of bound Cu in the normal active site produces a conformational change that exposes the hSOD1 epitope (aa 24-36). However, as the protein organizes into inclusions, this epitope becomes inaccessible, indicating that this portion of the protein must be buried within very stable structures in the inclusions.

An antibody termed C4F6, which was raised against the G93A variant of human SOD1, did not recognize inclusion structures formed by either untagged or YFP-tagged proteins. The epitope for the C4F6 antibody is complex. Under denaturing conditions, this antibody behaves as if the epitope includes the specific mutation of Gly 93 to Ala [27]. On denaturing immunoblots, C4F6 shows high specificity to the G93A variant with no reactivity to WT or other FALS associated mutations at Gly 93 [27]. However, under non-denaturing conditions, the antibody behaves as if the epitope includes conformational elements as the antibody can recognize other mutant forms of SOD1, albeit much less robustly [24]. The antibody shows no reactivity to native WT SOD1 [27]. Thus, although it is likely that the conformational elements recognized by this antibody are in proximity to the primary epitope that includes Gly 93, within exon 4, the epitope has not been precisely defined.

Our findings indicate that as mutant SOD1 assembles into inclusions, there is a conformational shift that either masks or destroys the C4F6 epitope. We note, however, that Okamato et al [39] reported that inclusions in the spinal cords of G93A mice were immunoreactive with the C4F6 antibody. It is possible that reactivity with inclusions in the G93A mice is unique to this model because the non-conformational elements of the epitope include the G93A mutation [27].

In our cell culture models, the SEDI and C4F6 antibodies showed the best discrimination between WT and mutant SOD1 in regard to the diffuse cytosolic staining. Although there were rare cells expressing WT SOD1 that showed reactivity, the frequency with which mutant cells were immunostained was much higher. The significance of the infrequent, but robust, staining of cells expressing WT SOD1 with these antibodies is uncertain. Bosco and colleagues recently reported that C4F6 will react with oxidatively modified WT SOD1 [27]. Thus, it is possible that some type of oxidative process in a subset of cells triggers the misfolding of WT SOD1. It is also possible that WT SOD1 can spontaneous misfold in rare cases of extreme over-expression with the misfolded conformation propagating throughout the cell.

In cells expressing the untagged A4V SOD1, both the C4F6 and SEDI antibodies robustly detected a diffusely distributed form of mutant SOD1 that was readily released from cells by saponin. We note that Zetterstrom and colleagues have demonstrated that spinal cord tissues from mice expressing both WT and mutant human SOD1 contain varied amounts (higher in mutant mice) of soluble SOD1 that aberrantly binds hydrophobic media and lacks normal intramolecular disulfide bonds [40]. Thus, there are multiple lines of evidence to indicate soluble malfolded forms of mutant protein exist at least transiently. Whether these soluble malfolded forms of mutant SOD1 are precursors of the aggregates that form or a misfolded soluble entity that is off pathway for aggregation is uncertain.

In comparing the C4F6 and SEDI antibodies, each have unique utility. The SEDI antibody would appear to be a very good antibody for labeling inclusion structures. Although this antibody is conformationally-restricted in reactivity as it does not recognize natively folded dimeric WT SOD1 [17], it is not a conformation specific epitope; this antibody will recognize denatured WT SOD1 and could presumably recognize an immature form of SOD1 in a monomeric state that is on pathway for native folding. In our hands, the C4F6 antibody seems to behave more like a conformation specific antibody in that it does not recognize denatured WT SOD1 or denatured forms of mutant SOD1, except for specific reactivity with denatured G93A SOD1 [27]. While this later finding would indicate reactivity with a specific mutant sequence of SOD1 (the G93A mutation and flanking sequence), the reactivity of C4F6 with diffusely distributed cytosolic SOD1 in cells expressing A4V SOD1 is consistent with the antibody recognizing a specific mal-folded conformation as opposed to simply recognizing an immature form of mutant SOD1 or a specific sequence epitope. Thus, the C4F6 antibody may be uniquely valuable in identifying mal-folded SOD1 proteins. Interestingly, Bosco et al described diffuse labeling of motor neurons of sporadic ALS cases with the C4F6 antibody, suggesting that misfolding of WT SOD1 occurs at some point in the course of sporadic disease [27].

We note, however, that Karumbayaram and colleagues used C4F6 in immunostains of ES cell-derived motor neurons expressing WT, A4V, I113T, and G93A mutants, finding that the antibody specifically recognized cells expressing the G93A variant [41](Additional File 1, Table S1). In this study, it appears that the sequence specific epitope of the C4F6 antibody predominated. The level of mutant SOD1 expression in these cells was not characterized and it is difficult to explain why the ES-motor-neuron cell model did not show the misfolding of other mutants that were expressed.

### Immunohistochemical analyses of transgenic mouse models and human ALS with conformationally restricted antibodies

The hSOD1 antibody has been extensively used in immunohistochemical analyses of spinal cord tissues from mice expressing WT and mutant SOD1 with the pattern of immunoreactivity showing some variability depending upon how the tissues were prepared (Additional File 1, Table S1). Generally, the antibody shows cell body reactivity that is similar to what we have described here in our cell model in tissues from mutant SOD1 transgenic mice when the tissues have been prepared for immunohistochemistry by cryoprotection and frozen sectioning, with no antigen retrieval (Additional File 1, Table S1). Immunolabeling of cell bodies in mutant mouse tissues prepared by paraffin embedding with microwave heating for antigen retrieval has generally been weak, but there are also examples of robust cell body immunoreactivity (Additional File 1, Table S1). The only published example of immunostaining of tissues from mice that over-express WT SOD1 utilized a paraffin embedding method with microwave heating for antigen retrieval, finding relative weak labeling of cell bodies with diffuse neuropil immunoreactivity [42]. Importantly, hSOD1 antibody has consistently been found to show little or no reactivity with inclusion structures (Additional File 1, Table S1).

The C4F6 and SEDI antibodies have also been used in studies of tissues from human ALS and SOD1 mouse models, though not as extensively in the case of the C4F6 antibody. Okamato reported intense immunoreactivity in cell bodies of the G93A mice with the C4F6 antibody, with labeling of structures interpreted as inclusions [39](Additional File 1, Table S1). The degree to which this pattern of staining reflects the mutation specific component (G93A) of the C4F6 epitope is uncertain. Our data would indicate that C4F6 does not readily recognize inclusions formed by other fALS variants of SOD1. The SEDI antibody has been used in studies of mice expressing the G37R, G85R, and G93A mutants as well as human fALS and sALS cases [17](Additional File 1, Table S1). In the G85R mice and human fALS associated with A4V, A4T, I113T, V14M, and dG27/P28 mutations, SEDI antibody showed intense reactivity to inclusion structures in cell bodies. However, in mice expressing the G37R and G93A mutants, SEDI antibody primarily immunostained the margins of vacuolar pathology that is prevalent in these animals [17]. If inclusions were also present in the spinal cords of these mice, they were not selectively recognized.

Overall, these findings are consistent with our results and indicate the utility of the SEDI antibody in detecting mutant SOD1 inclusion pathology. It is noteworthy that spinal cord tissues from sALS patients lacking mutations in SOD1 show little or no reactivity with the SEDI antibody [43]. However, Forsberg and colleagues reported that an SOD1 antibody raised against a C-terminal peptide that included the epitope of the SEDI antibody immunostained small punctate inclusion structures in motor neurons of sALS cases as well as non-SOD1 fALS cases [44]. Whether differences in antibody specificity or differences in tissue preparation account for the different outcomes is unknown and the potential involvement of misfolded WT SOD1 within motor neurons in sALS remains a debated topic.

### Aggregation and toxicity

In the cell models we use here to examine mutant SOD1 folding, we observe very low levels of cell death by the methods we used to detect dying cells. This finding indicates that misfolded and aggregated forms of mutant SOD1 are not acutely toxic in these cell models. We focused effort on models in which SOD::YFP fusion proteins were expressed so that we could definitively identify transfected cells. Cells harboring inclusions generally lacked evidence of impending death and very little evidence of toxicity was noted in cells showing more diffuse distributions of SOD::YFP fusion proteins. Although, it is possible that cells that died detached from the plate quickly and escaped detection by our methods, we are left with no clear indication of toxicity.

Several previous studies have sought to determine whether aggregates of mutant SOD1 are associated with toxicity in a variety of neural cell models. We are aware of only two studies that have used methods that would allow for simultaneous assessment of aggregation (via expression of YFP fusion proteins) and visual reporters of cell toxicity. Using a rat PC12 cell model, which can be differentiated into neuron-like cells, and time-lapse analysis, Matsumoto et al [31] observed that cells that formed inclusions of SOD1::YFP fusion proteins were much more likely to die. However, cell death was also observed in cells lacking inclusions. Zhang and Zhu examined toxicity in NSC34 cells, which were created by fusion of murine neuroblastoma cells and murine motor neurons [45], finding no evidence for direct toxicity despite the presence of aggregates in cells expressing mutant SOD1 fused to GFP [29]. In this study, NSC34 cells stably expressing mutant SOD::GFP fusion proteins were reported to be more sensitive to oxidative stress induced by low levels of H_2_O_2_, but there was no reported examination of whether cells with inclusions were specifically more sensitive. A similar lack of direct toxicity by mutant SOD1 fused to eGFP when expressed in NSC34 cells was reported by Turner et al. [33].

There are two reports describing the expression of mutant SOD1 fused to GFP in mouse neuroblastoma N2a cells. One study reported finding a correlation between expression of mutants that readily form inclusions and toxicity (MTT assay) [46]. In this study, experimental mutations that changed cysteine residues at position 6 and 111 reduced aggregation and reduced toxicity. In a second study, Witan et al [47] failed to observe direct toxicity due to expression of mutant SOD1 fusions with EGFP despite detection of inclusions at high frequency. However, similar to Zhang and Zhu [29], Witan et al reported that cells expressing the mutant fusion proteins were more sensitive to oxidative stress inducing agents [47]. The authors did not investigate whether cells with inclusions were specifically more sensitive to oxidative stress. The study by Witan differs from all others in that the fusion proteins expressed were engineered as contiguous homodimers or contiguous heterodimers (mutant with WT). Overall, the outcomes of these investigations provide a mixed view of the role of mutant SOD1 aggregation in toxicity, and it remains difficult to explain divergent outcomes.

In previous work in transgenic mice there has been much effort to define changes in the physical nature mutant SOD1 through the course of disease. In an elegant study by Wang and colleagues, expression of a G85R variant of human SOD1 fused to YFP clearly demonstrated that large visible inclusions form very late in the course of disease [48]. In our previous studies of transgenic mouse models of ALS expressing untagged variants of mutant SOD1, we have similarly observed that the greatest accumulation of detergent-insoluble aggregates of mutant SOD1 occurs late in disease progression [22]. In the G93A-Gur1 model, which reaches end-stage paralysis at about 120 days, aggregates begin to accumulate between 90 and 105 days and then rise dramatically as the animals approach endstage [22]. During this same interval, serum levels of neurofilament H, which serve a biomarker of axonal degeneration [23], similarly rise dramatically. Thus we can correlate aggregation of mutant SOD1 and axonal degeneration in the animal models. Moreover, in the G93A model, the majority of motor neuron death occurs during the interval in which detergent-insoluble aggregates appear.

Although it is tempting to link inclusion formation to motor neuron death, it remains difficult to assign mutant protein aggregation as causative of cell death as it is possible that the accumulation of aggregates serves as a biomarker of cellular degeneration in which the cells simply lose the ability to prevent the misfolded mutant protein from aggregating because of some combination of declining chaperone activities, declining proteasome activity to degrade the misfolded protein, or declining energy production to support these activities. It may not be possible to sort out the role of aggregation in toxicity until we have tools to prevent such aggregates from occurring in vivo [49].

Although we might wish to be able to assign toxicity to a specific conformation, assembly, or aggregate of mutant SOD1, it seems likely that multiple forms of misfolded SOD1 exert deleterious effects. For example, it is fairly well established that early, during the insidious phase of disease when pathologic abnormalities are observed with limited symptoms, there is limited accumulation of the detergent-insoluble aggregates; instead, detergent soluble forms of mutant SOD1 predominate [22]. As mentioned above, a previous study by Zetterstrom et al demonstrated that early in the course of disease, spinal cords of mice expressing mutant SOD1 contain soluble forms of mutant SOD1 that bind to hydrophobic affinity chromatography media and lack the normal intramolecular disulfide linkage [40]. Whether these non-natively folded forms of mutant SOD1 mediate early events in toxicity remains to be established, their early appearance in tissues from the mutant mice clearly makes them candidates. Our findings indicate that the C4F6 antibody might be useful in detecting soluble misfolded forms of mutant SOD1 early in the course of disease. However, we would recommend that such studies focus on mutants other than the G93A mutant to avoid mutation specific reactivity in favor of misfolded confirmation reactivity. It would, of course, be easier to explain the disease if one specific form of mutant SOD1 (monomer, oligomer, aggregate, or inclusion) targeted one specific process that is unique to neurons and of greater importance to motor neurons, but the available evidence suggests multiple systems may be targets of mutant SOD1 toxicity [50].

## Conclusions

The present study uses a combination of natural SOD1 mutant and SOD1::YFP fusion proteins to examine the relationships between detergent insoluble structures, aggregates, and inclusion body formation. Studies in mice, using both biochemical methods of detection and YFP tagging methods have established that larger aggregates form late in the disease, primarily during the time of robust cell death and rapid disease progression [22,48]. However, these studies also establish that pathologic and behavioral symptoms occur in mice prior to the accumulation of these larger aggregates, implying that smaller assemblies of mutant SOD1 must mediate some aspect of toxicity and trigger disease. Our studies define the utility of different approaches in detecting mal-folded forms of WT and mutant SOD1 that are both soluble and insoluble. Going forward, it may be possible through the use of these various tools to better define the role of these various assemblies of mutant SOD1 in the evolution of pathologic and behavioral abnormalities that define the ALS mouse models.

## Methods

### SOD1 cDNA expression plasmids and cell lines

WT and mutant hSOD1 untagged proteins were expressed from plasmids based on the mammalian pEF-BOS expression vector, and have been previously described [1,16,20,21]. *YFP *tagged SOD1 cDNA variants were created from a worm expression vector (pPD30.38) that contains WT hSOD1 fused to eYFP (yellow fluorescent protein) kindly provided by Dr. Rick Morimoto (Northwestern University). WT::YFP, WT::RFP and YFP variants have been previously described [35], and mutant fluorescently tagged SOD1 variants were constructed following similar procedures.

All cell culture studies of SOD1 aggregation used HEK293FT cells (Invitrogen, Carlsbad, CA), mouse TK-L cells, or CHO cells (ATCC, Manassas, VA) as indicated, which were maintained following ATCC recommendations.

### Transfections

Transfection of cells for immunocytochemistry was performed on glass coverslips that were previously coated with 0.5 mg/ml poly-L-lysine in 1× phosphate buffered saline solution (PBS). A total of 2 μg of vector DNA was transfected per well, using Lipofectamine 2000 (Invitrogen, Carlsbad, CA). For biochemical analyses, a total of 4 μg of vector DNA was used to transfect cells in 60 mm poly-L-lysine coated dishes (BD Biosciences, Bedford, MA). Each transfection experiment was repeated a minimum of 3 times.

### Immunocytochemistry

Transfected cells were either fixed with 4% paraformaldehyde in 1× PBS solution for 15 minutes or incubated for 30 minutes in mild detergents (0.01% digitonin or 0.01% saponin in 1× PBS) prior cell fixation. Cells were then completely permeabilized using cold 100% methanol for 5 minutes followed by incubation in 20% normal goat serum in 1× PBS and immunostaining with hSOD1 (1:500), SEDI (1:500), or C4F6 (1:500) antibodies overnight (in 1× PBS with 10% normal goat serum). Primary antibodies were visualized with secondary fluorescently-labeled antibodies (1:2000) diluted in same buffer as the primary antibody (Alexafluor goat anti-rabbit 594 nm for hSOD1 or SEDI, or Alexafluor goat anti-mouse 568 nm for C4F6 antibody; Invitrogen, Carlsbad, CA). To visualize nuclei, cells were treated with DAPI solution (4',6-diamidino-2-phenylindole, dihydrochloride, stock 14.3 mM from Invitrogen, Carlsbad, CA) at a dilution of 1:2000 for 10 minutes. Fluorescence was visualized using an Olympus IX81-DSU spinning disk confocal microscope or an epifluorescence Olympus BX60 microscope as indicated in the Figure Legends.

### Detergent extraction of SOD1 aggregates

Extraction of cultured cells in nonionic detergents (NP-40) was performed as described previously [1,21,22]. The procedure produces two protein fractions termed S1 (detergent-soluble) and P2 (detergent-insoluble), which were then analyzed by immunoblotting (5 μg total protein from S1 is compared to 20 μg total protein from P2). We then quantified the intensity of the SOD1 bands in each fraction and calculated the mean ratio of insoluble to soluble protein. Data were then analyzed on GraphPad PRISM 5.01 Software (La Jolla, CA) to determine statistical differences, using unpaired student *t*-tests, and generate graphic representations.

### Cell Death Assays

To assess nuclear DNA fragmentation, we used TUNEL labeling assays (In Situ Cell Death Detection Kit, Roche Applied Science, cat# 12 156 792 910, Indianapolis, IN, USA). Briefly, CHO cells were plated into 12-well plates containing lysine-coated coverslips and grown overnight. The cells were then transfected with 0.8 μg DNA in 2 μl of Lipofectamine 2000 diluted, ultimately in 500 μl of Opti-MEM. After 3 h at 37°C, 500 μl of complete media (Ham's F12 with 10% fetal bovine serum and 2 mM L-glutamine) was added. For a positive control, one well of untransfected cells was treated with staurosporine 1 or 2 μM final concentration, (40 μl or 80 μl of a 50 mM stock in 1 ml media) overnight at 37°C. Twenty-four hours after transfection, cells were rinsed 3 times with 1× PBS, fixed in 4% paraformaldehyde in 1× PBS for 20 min at RT, and then rinsed 3 times in 1× PBS. Before immunostaining, the cells were permeabilized in pre-heated 10 mM Tri-sodium citrate and 0.05% Tween 20 (pH 6.0) for 10 min at 90 C, and then rinsed 3 times in 1× PBS.

For TUNEL labeling, we followed the manufacturer's protocol, which entails making a reaction mixture by mixing 8 μl of enzyme solution into a 72 μl labeling solution that is kept in the dark on ice until ready to use. Each coverslip was then incubated in the labeling solution by pipetting 18 μl of the solution mix onto half of the coverslips, which was then covered with a piece of parafilm to keep it from drying out. The other half of the coverslips were covered 18 μl of labeling solution with no enzyme for a negative control (also covered with parafilm). All coverslips were incubated in a humid chamber at 37°C for 2 - 2.5 hours before rinsing 3 times with 1× PBS followed by floating off and removing the parafilm. Coverslips were then mounted on glass slides using Aqua-Poly/Mount (Polysciences, Inc., Warrington, PA, USA, cat # 18606) and photographed.

A second assay for cell death utilized the LIVE/DEAD Viability/Cytotoxicity Kit for mammalian cells [Invitrogen (Molecular probes), Carlsbad, CA, USA, cat # L3224]. For this assay, cells were grown on coverslips and transfected as described above. Positive control cells, treated with staurosporine, were also generated as described above. After 24 or 48 hours of incubation, the cells were rinsed 3 times with 1× PBS and then incubated with Component B of the kit (Ethidium homodimer-1) at 5 or 10 μM in 1× PBS at room temperature or 37°C for 10 to 30 minutes. The coverslips were then rinsed in 1× PBS, mounted on glass slides and sealed using clear nail polish before photography. We also found that after removal of excess reagent, the cells could be fixed in 4% paraformaldehyde and visualized.

## Competing interests

The authors declare that they have no competing interests.

## Authors' contributions

MP and DRB designed research, MP performed research, MP and DRB oversaw the effort of technical assistants named below, analyzed data. DRB and MP wrote the manuscript. All authors read and approved the final manuscript.

## Supplementary Material

Additional file 1 **Supplementary Figures S1-8; Supplementary Table 1**. Suppl. Figure 1. The majority of SOD1 in transiently-transfected HEK293FT transfected cells is soluble in non-ionic detergent. Suppl. Figure 2. Formic acid treatment does not allow visualization of inclusions using a hSOD1 antibody. Suppl. Figure 3. TK negative cells transfected with SOD1 constructs for 48 hours and stained for human SOD1. Suppl. Figure 4. HEK293FT cells expressing a SOD1::YFP variant accumulate detergent-insoluble aggregates. Suppl. Figure 5. Digitonin treatment in TK negative cells reveals a punctate pattern of SOD1 immunoreactivity in cells expressing either WT or A4V SOD1. Suppl. Figure 6. Digitonin extracts a portion of the detergent-soluble hSOD1 protein expressed in HEK293FT cells. Supplemental Figure 7. EtBr uptake assay of cell permeability in HEK293FT cells expressing mutant and WT YFP fusion proteins. Suppl. Figure 8. Assessment of cell death in CHO cells expressing WT and A4V SOD::YFP fusion proteins. Supplementary Table 1. Summary of previous findings with conformationally-restricted antibodies [4,5,15,17,26,27,39,41-43,51-56]. Click here for file
