# Supplementary material for: Superoxide dismutase 1 encoding mutations linked to ALS adopts a spectrum of misfolded states
Source: Mol Neurodegener. 2011 Nov 17;6:77. doi: 10.1186/1750-1326-6-77 (PMC3248846; doi:10.1186/1750-1326-6-77)
Supplement: Additional file 1 — Supplementary Figures S1-8; Supplementary Table 1. Suppl. Figure 1. The majority of SOD1 in transiently-transfected HEK293FT transfected cells is soluble in non-ionic detergent. Suppl. Figure 2. Formic acid treatment does not allow visualization of inclusions using a hSOD1 antibody. Suppl. Figure 3. TK negative cells transfected with SOD1 constructs for 48 hours and stained for human SOD1. Suppl. Figure 4. HEK293FT cells expressing a SOD1::YFP variant accumulate detergent-insoluble aggregates. Suppl. Figure 5. Digitonin treatment in TK negative cells reveals a punctate pattern of SOD1 immunoreactivity in cells expressing either WT or A4V SOD1. Suppl. Figure 6. Digitonin extracts a portion of the detergent-soluble hSOD1 protein expressed in HEK293FT cells. Supplemental Figure 7. EtBr uptake assay of cell permeability in HEK293FT cells expressing mutant and WT YFP fusion proteins. Suppl. Figure 8. Assessment of cell death in CHO cells expressing WT and A4V SOD::YFP fusion proteins. Supplementary Table 1. Summary of previous findings with conformationally-restricted antibodies [4,5,15,17,26,27,39,41-43,51-56]. [file 1750-1326-6-77-S1.PDF]

Suppl. Fig. 1.

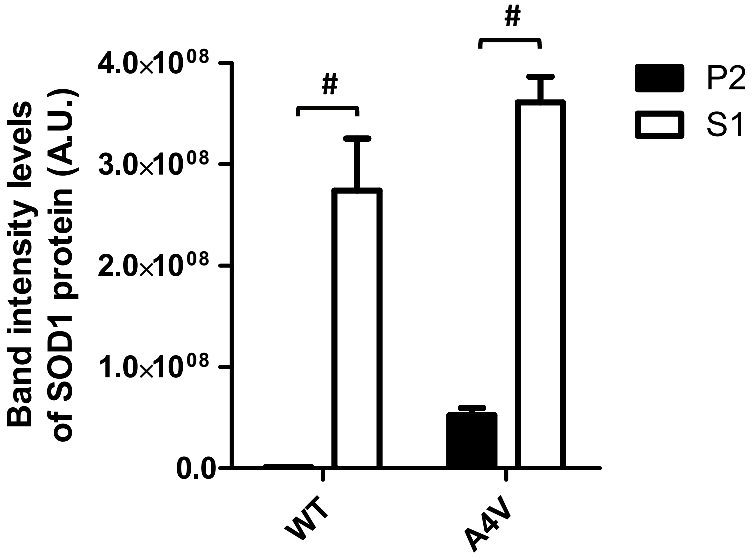

Suppl. Fig. 1. The majority of SOD1 in transiently-transfected HEK293FT transfected cells is soluble in non-ionic detergent. Band intensities were calculated from western blots of P2 (black bars) and S1 (white bars) fractions, and adjusted for the amount of total protein loaded on the SDS-PAGE gels (5 μg for S1 and 20 μg for P2). The data represented here derives from at least five independent transfection experiments. Significant differences exist between the levels of SOD1 P2 *vs*. S1 fractions for each SOD1 protein expressed; unpaired student *t*-test: $^{\#}p \leq 0.005$. The data were averaged from 3 independent transfection experiments.

A possible explanation for the inability to visualize inclusions in transiently-transfected cells may be related to the amount of detergent-soluble SOD1 protein *vs.* the amount of detergent-insoluble aggregated SOD1 protein that is present. In transient 24 hour transfections, the levels of detergent-soluble SOD1 protein in HEK293FT cells expressing mutant A4V SOD1 is about 4 times higher than the levels of detergent-insoluble aggregated proteins. Thus, it seems reasonable to explain our inability to observe inclusions may be due to a masking effect in which soluble protein obscures visualization of inclusions.

Suppl. Fig. 2.

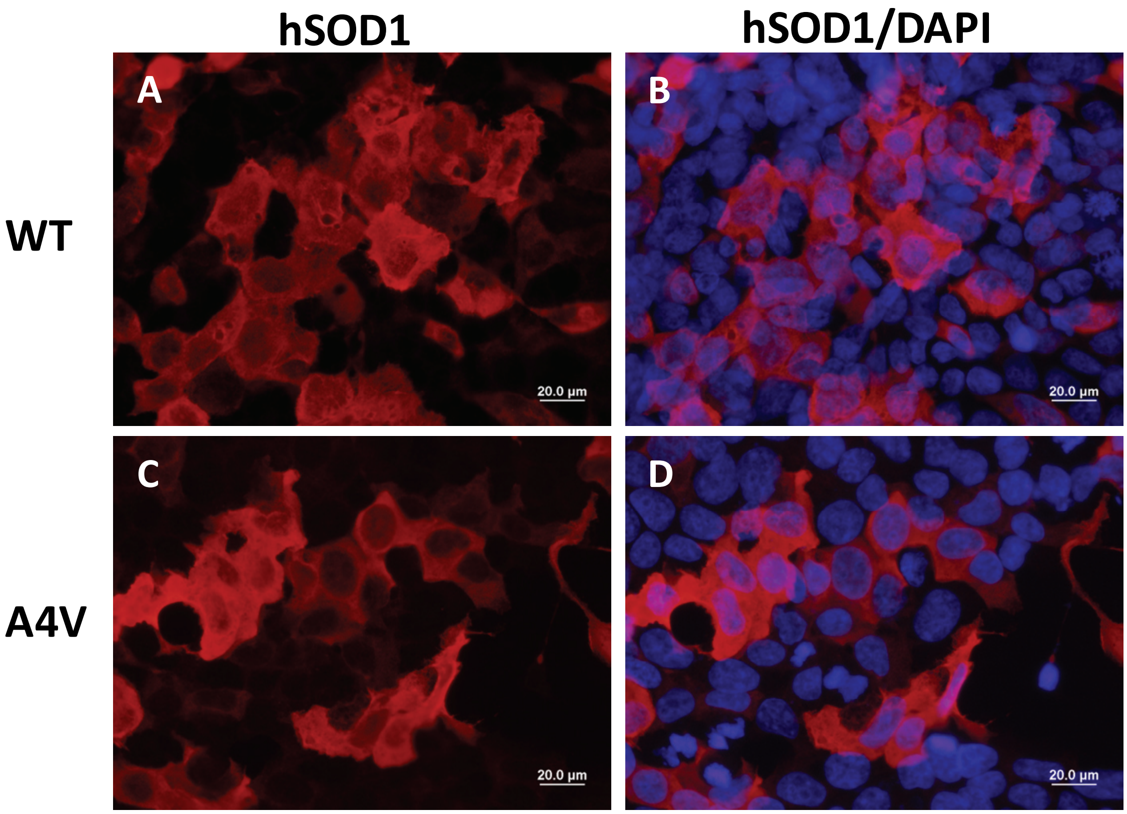

Suppl. Fig. 2. Formic acid treatment does not allow visualization of inclusions using a hSOD1 antibody. HEK293FT cells were transfected with either WT (A-B) or A4V (C-D) hSOD1 for 24 hours. Staining with hSOD1 antibody was performed as described in Methods, but here cells were treated with 70% formic acid for 10 minutes prior to fixation of the cells. Images were captured with a 40x objective. Scale bars = 20 µm. The images shown are representative of at least 3 independent transfection and immunostaining experiments.

Suppl. Fig. 3.

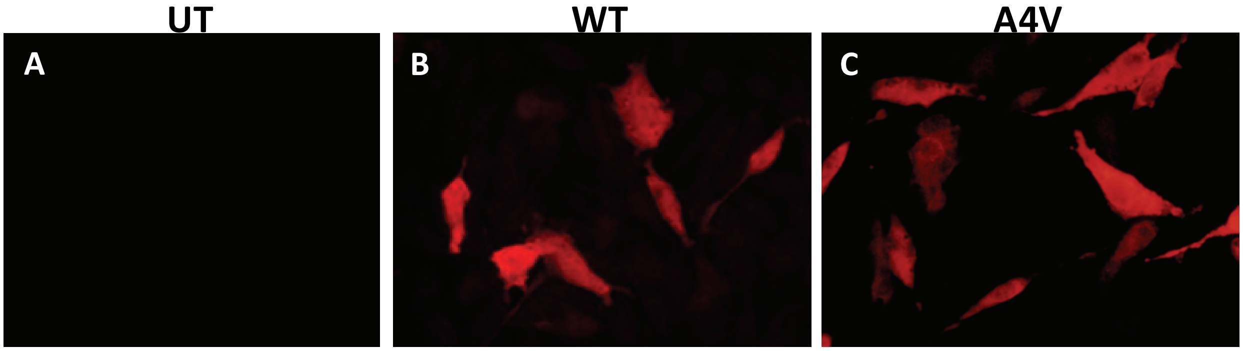

Suppl. Fig. 3. TK negative cells transfected with SOD1 constructs for 48 hours and stained for human SOD1 as explained in Methods. Pictures were taken with a 40x objective. UT denotes untransfected cells.

A possible explanation for our inability to detect mutant SOD1 inclusions resides in the possibility that the amount of endogenous WT SOD1, produced by the HEK293FT cells, may increase the overall hSOD1 staining and interfere with the visualization of protein inclusions. Thus, to eliminate the background generated by endogenous WT SOD1 protein, we performed transient transfections in a mouse fibroblast cell line (TK- L cells) and used antibodies specific to human SOD1 to detect transfected protein. In mouse TK- L cells, we do not detect detergent-insoluble SOD1 aggregates biochemically until 48 hours post-transfection (data not shown). Therefore, we immunostained fixed cells at 48 hrs, finding WT and A4V SOD1 proteins showed a similar diffuse pattern of fluorescence. The images shown are representative of at least 3 independent transfection and immunostaining experiments.

Suppl.Fig. 4.

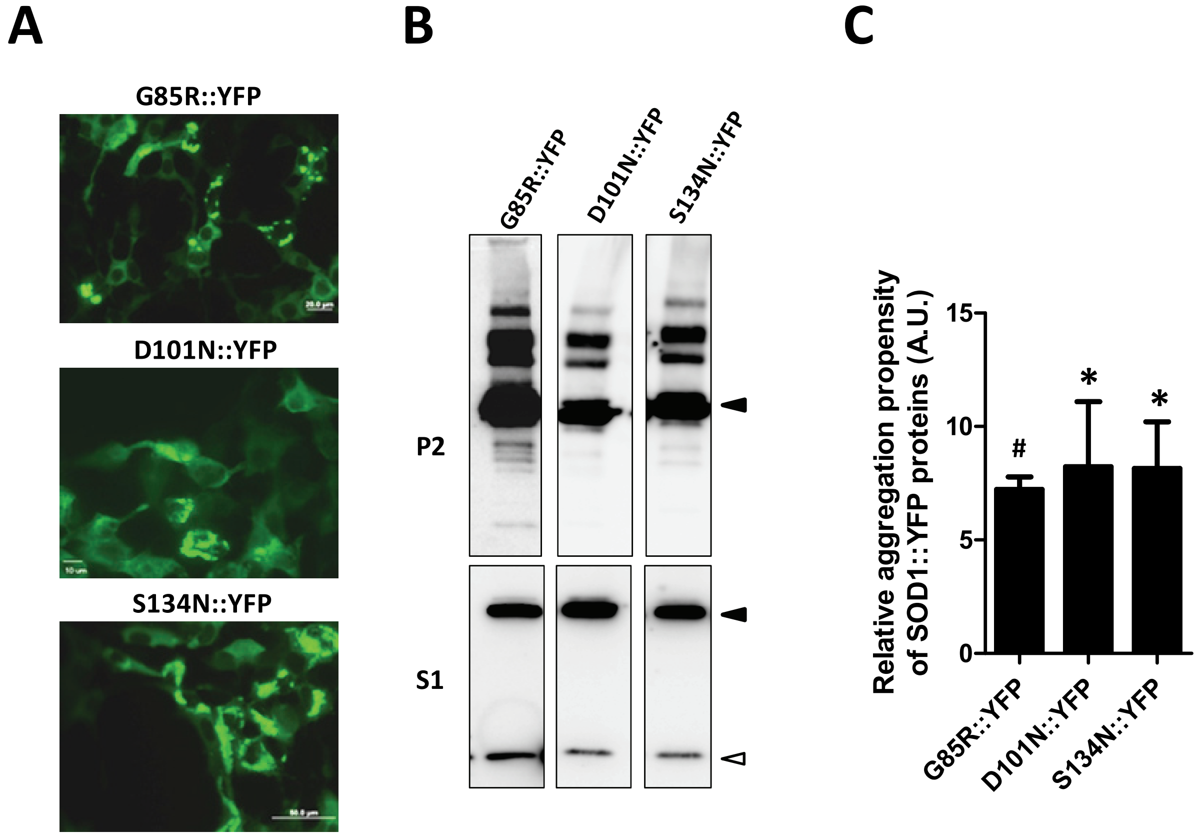

Suppl. Fig. 4. HEK293FT cells expressing a SOD1::YFP variant accumulate detergent-insoluble aggregates. A) Fluorescence images of SOD1::YFP constructs expressed for 24 hours. B) Immunoblot of P2 and S1 protein fractions of cells expressing SOD1::YFP proteins for 24 hours. The SOD1::YFP protein migrates at a relative molecular mass of 50 kDa (filled arrowhead), while endogenous WT SOD1 monomer migrates at 16 kDa (open arrowhead). C) Quantification of the aggregation propensity (P2/S1) of cells expressing SOD1::YFP proteins. The data were averaged from at least 3 independent transfection experiments for each mutant. Unpaired student *t*-tests were performed to compare the level of detergent-insoluble mutant SOD1::YFP with that of WT::YFP SOD1 fusion protein (see Fig. 1 for immunoblot of WT::YFP): $*p \leq 0.05$; $^{\#}p \leq 0.005$.

Suppl. Fig. 5.

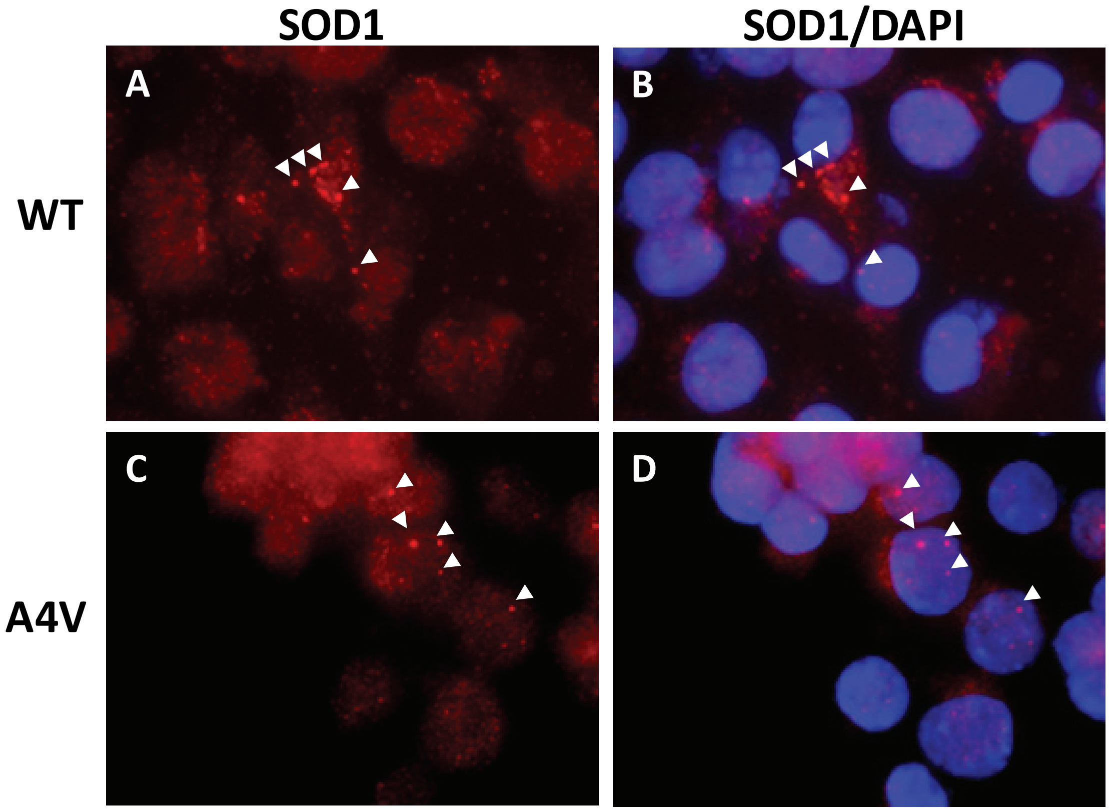

Suppl. Fig. 5. Digitonin treatment in TK negative cells reveals a punctate pattern of SOD1 immunoreactivity in cells expressing either WT or A4V SOD1. To test whether membrane permeabilization of transfected TK- L cells would release soluble SOD1 and reveal inclusions, we treated cells with digitonin (similar in properties to saponin) at 48 hours post-transfection. In these cells, we observed hSOD1 to immunostains small punctate structures in cells expressing both WT and A4V mutant SOD1. No obvious difference in the frequency or intensity of these structures as noted. A-D) TK- L cells were transfected for 48 hours, treated with 0.01% digitonin in 1x PBS, and stained for human SOD1. Transfection, detergent treatment and staining were performed as described for HEK293FT cells in Materials and Methods. Images corresponding to WT (A-B) and A4V (C-D) were taken using an immersion oil 100x objective. The images shown are representative of at least 3 independent repetitions of the experiment.

Suppl. Fig. 6.

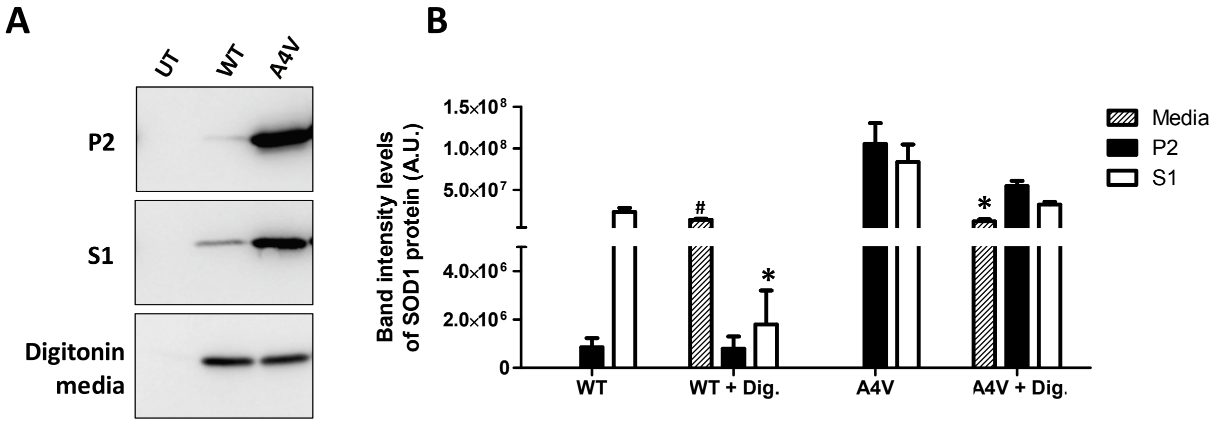

Suppl. Fig. 6. Digitonin extracts a portion of the detergent-soluble hSOD1 protein expressed in HEK293FT cells. Digitonin released WT SOD1 into medium with a corresponding decrease in the amount of WT SOD1 in the S1 fraction of digitonin treated cells. In cells expressing A4V SOD1, digitonin released mutant protein into the media, but the level of mutant protein in the S1 and P2 fractions of treated cells was not statistically different from that of untreated cells. A) Immunoblot of HEK293FT cells transfected with the indicated hSOD1 constructs for 48 hours. Prior harvest, cells were incubated 30 minutes in 1x PBS with 0.01% digitonin (Dig.). Then the harvested cell pellets were analyzed by detergent extraction and centrifugation assays. The amount of hSOD1 protein released into the cell media was also evaluated. B) Quantification of SOD1 protein found in the Media, P2 and S1 fractions of cells. Symbols over bars indicate statistical differences from the corresponding untreated control. The data were averaged from 3 independent transfection experiments for each construct and condition. Unpaired *t*-tests: $*p \leq 0.05$; $^{\#}p \leq 0.005$.

Suppl. Figure 7

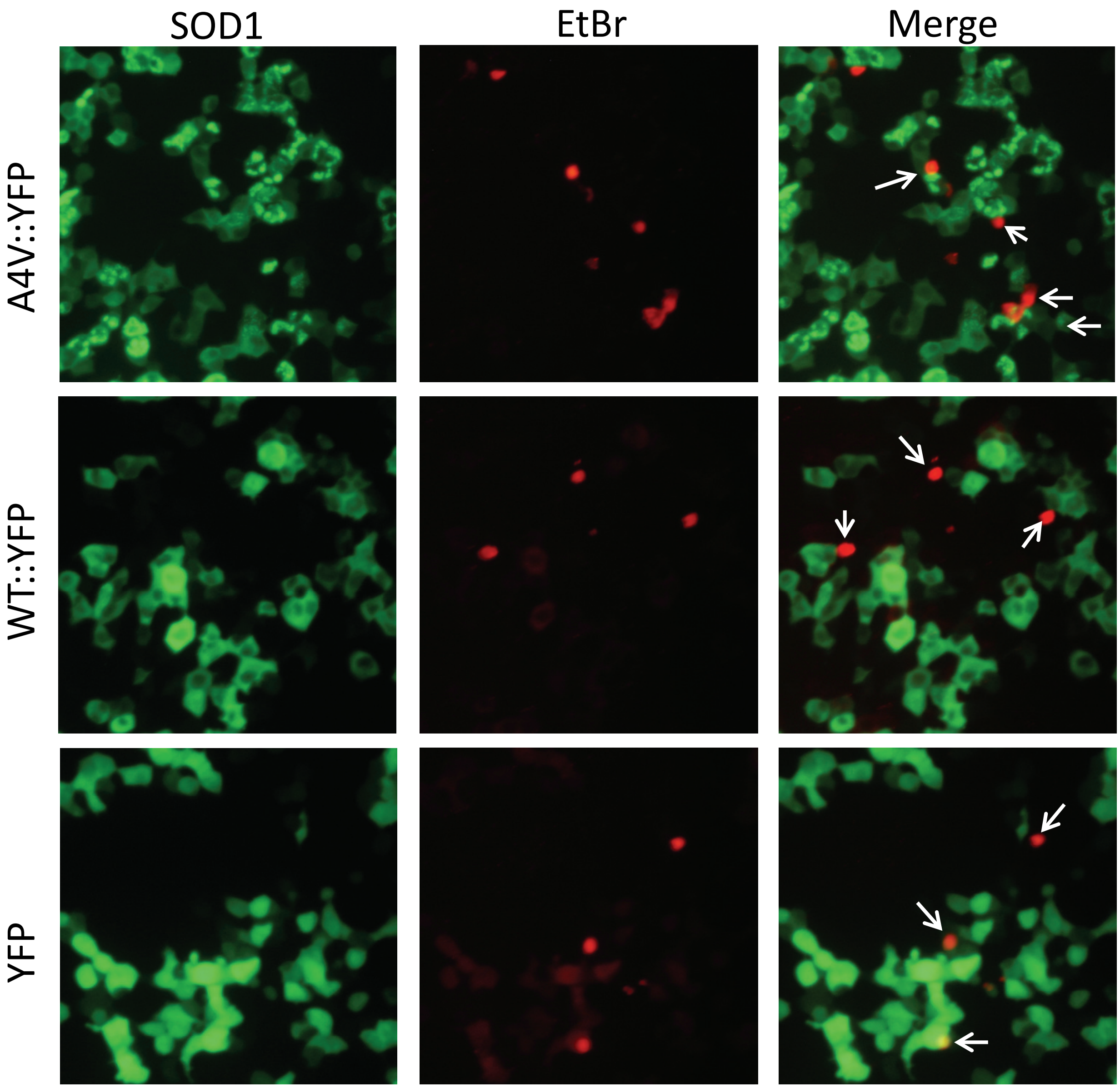

Supplemental Figure 7. EtBr uptake assay of cell permeability in HEK293FT cells expressing mutant and WT YFP fusion proteins. Cells were transfected for 24 hours, and then incubated with the EtBr cell permeability reagent (see main text). Images were captured on live cells on an inverted fluorescence microscope at 40X.

Suppl. Fig. 8.

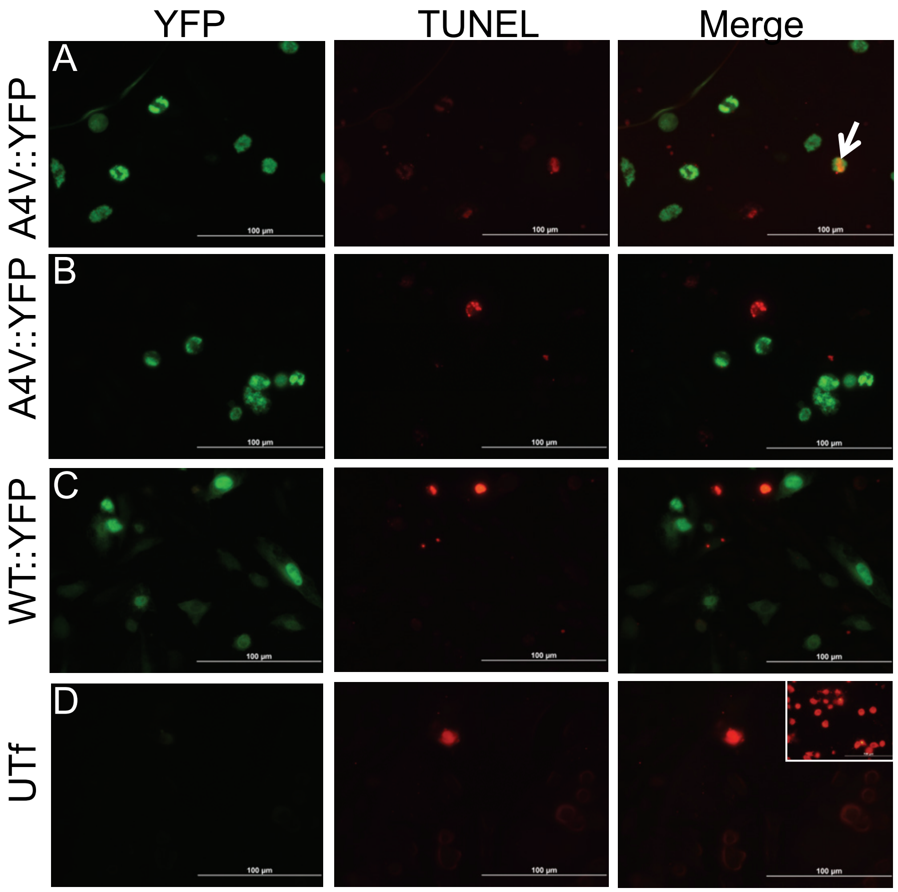

Suppl. Fig. 8. Assessment of cell death in CHO cells expressing WT and A4V SOD::YFP fusion proteins. At 48 hours post-transfection, we found that relatively few cells were TUNEL positive and only rarely did we observe labeling of cells that expressed A4V:YFP SOD1 fusion proteins (Rows A and B). A similar low frequency of TUNEL positive cells were seen in cells expressing WT::YFP SOD1 fusion proteins (Row C) and untransfected cells (Row D). As a positive control, cells treated with staurosporine demonstrated robust TUNEL labeling (Row D, inset far right panel).

Supplementary Table 1. Summary of findings with conformationally-restricted antibodies.

Antibody	Model	Technique	Findings	Reference
hSOD1	G93A	Frozen, 4% paraformaldehyde, No antigen retrieval	Intense labeling of motor neuron cell bodies and neuritis in gray matter.	[51]
hSOD1	G37R, L126Z	Frozen, 4% paraformaldehyde, No antigen retrieval	Intense labeling of motor neuron cell bodies and neuritis in gray matter.	[26]
hSOD1	H46R/H48Q, WT	Paraffin, 4% paraformaldehyde, antigen retrieval - microwave heat	In both mutant and WT mice, diffuse labeling of neuropil, no obvious labeling of cell bodies. Weak labeling of inclusion-like structures in mutant mice.	[42]
hSOD1	PrP vector G37R mice	Paraffin, 4% paraformaldehyde, antigen retrieval - microwave heat	In young presymptomatic mice, intense labeling of motor neuron cell bodies, neuritis of neuropil.	[52]
hSOD1	L126Z	Paraffin, 4% paraformaldehyde, antigen retrieval - microwave heat; Frozen, 4% paraformaldehyde,	Progressive accumulation of immunoreactivity in cell bodies and neuritis of neuropil.	[53]
C4F6	Human sALS	Paraffin, fixation unknown	Intense labeling of motor neuron cell bodies, some cases of neuropil labeling.	[27]
C4F6	hESC-derived motor neurons	In situ preparation	Cells expressing G93A labeled – showing punctate structures. No labeling of cells expressing WT, A4V, or I113T mutants.	[41]

C4F6	G93A mouse	Frozen, 4% paraformaldehyde, No antigen retrieval described	Intense labeling of cell bodies and neuropil. Some structures reported to resemble inclusions.	[39]
SEDI	G37R , G85R, G93A mice,<br><br>Human A4V fALS	Paraffin, 4% paraformaldehyde,<br><br>Peroxide/citrate pretreatment, no heat	Mice - Intense immunoreactivity at margins of vacuolar pathology in G37R and G93A mice. Reactivity with inclusion structures in G85R mice. Overall reactivity with cell bodies limited in all cases.<br><br>Humans – Examples of reactivity in cell body, round inclusions in neuropil.	[17]
SEDI	Human SOD1-fALS, nonSOD1-fALS, sALS	Paraffin, formalin, Antigen retrieval - Citrate with heat	Intense immunoreactivity with inclusion structures of motor neurons from SOD1-linked fALS cases (A4V, I113T, A4T, deleted G27/P28, V14M). No reactivity in ALS patients lacking SOD1 mutations.	[43]
m/h SOD1	G37R	Paraffin, 4% paraformaldehyde, antigen retrieval - microwave heat	Diffuse neuropil labeling, intensification around vacuoles, some cell body labeling, rare inclusion-like structures.	[15,54]
m/h SOD1	NonTg mouse	Frozen, 4% paraformaldehyde,<br><br>No antigen retrieval	Intense labeling of cell bodies, neurites.	[55]

m/h Sod1	G37R, WT	Paraffin, 4% paraformaldehyde, antigen retrieval - microwave heat	In G37R mice – intense labeling at margins of vacuolar pathology. White matter tracts labeled. Limited labeling of grey matter neuropil or cell bodies.<br><br>In WT over-expressing mice- General neuropil labeling, intensification of white-matter tracts. Limited intensification of cell bodies as compared to non-transgenic animals	[56]
m/h SOD1	G85R	Paraffin, 4% paraformaldehyde, antigen retrieval – microwave heat	Intense reactivity with inclusions in motor neuron cell bodies.	[4]
m/h SOD1	G85R and G93A mice; human SOD1-fALS –L126 truncation	Paraffin, 4% paraformaldehyde, antigen retrieval – microwave heat	Mice – intense reactivity with inclusions in motor neuron cell bodies and neuropil.<br><br>Human – increase reactivity with neuropil inclusions	[5]
